# A Camera-Trap Survey of Mammals in Thung Yai Naresuan (East) Wildlife Sanctuary in Western Thailand

**DOI:** 10.3390/ani13081286

**Published:** 2023-04-09

**Authors:** Supagit Vinitpornsawan, Todd K. Fuller

**Affiliations:** 1 Department of Environmental Conservation, University of Massachusetts, Amherst, MA 01003, USA; 2 Wildlife Conservation Office, National Parks, Wildlife and Plant Conservation Department, Bangkok 10900, Thailand

**Keywords:** conservation status, endangered species, Western Forest Complex, occurrence, photo-rates, tropical diversity

## Abstract

**Simple Summary:**

We used camera traps to survey wild mammals in a portion of western Thailand and recorded 32 species, 17 of which were IUCN-listed from Near Threatened to Critically Endangered. Of all photo-captures, 4 species comprised 62%, and several species were captured only rarely. This area holds a rich community of mammals, but some differences in photo-rates from an adjacent sanctuary and comparisons with other research on local mammals suggest that some species were missed because of their rarity and the limitations of the technique used.

**Abstract:**

The Thung Yai Naresuan (East) Wildlife Sanctuary (TYNE), in the core area of the Western Forest Complex of Thailand, harbors a diverse assemblage of wildlife, and the region has become globally significant for mammal conservation. From April 2010 to January 2012, 106 camera traps were set, and, in 1817 trap-nights, registered 1821 independent records of 32 mammal species. Of the 17 IUCN-listed (from Near Threatened to Critically Endangered) mammal species recorded, 5 species listed as endangered or critically endangered included the Asiatic elephant (*Elephas maximus*), tiger (*Panthera tigris*), Malayan tapir (*Tapirus indicus*), dhole (*Cuon alpinus*), and Sunda pangolin (*Manis javanica*). The northern red muntjac (*Muntiacus vaginalis*), large Indian civet (*Viverra zibetha*), Malayan porcupine (*Hystrix brachyuran*), and sambar deer (*Cervus unicolor*) were the most frequently recorded species (10–22 photos/100 trap-nights), representing 62% of all independent records, while the golden jackal (*Canis aureus*), clouded leopard (*Neofelis nebulosa*), marbled cat (*Pardofelis marmorata*), and Sunda pangolin were the least photographed (<0.1/100 trap-nights). Species accumulation curves indicated that the number of camera trap locations needed to record 90% of taxa recorded varied from 26 sites for herbivores to 67 sites for all mammals. TYNE holds a rich community of mammals, but some differences in photo-rates from an adjacent sanctuary and comparisons with other research on local mammals suggest that some species are rare and some are missed because of the limitations of our technique. We also conclude that the management and conservation plan, which involves the exclusion of human activities from some protected areas and strict protection efforts in the sanctuaries, is still suitable for providing key habitats for endangered wildlife populations, and that augmented and regular survey efforts will help in this endeavor.

## 1. Introduction

Mammals play a key role in the tropical ecosystem as modifiers of vegetation structure, seed dispersers, prey, and agents of change of species composition [1,2]. This role makes them prime candidates as indicator species for conservation, because the protection of large mammal species and their habitats also conserves a large part of the remaining community [3]. Even with these important roles, the status, occurrence, and abundance of mammals in many regions are understudied, with little data available for many species [3]. Moreover, habitat alteration, illegal hunting, and overall degradation of habitat due to human activities also has contributed to serious declines in populations [4,5,6]. Therefore, understanding species occurrence and relative abundance provides important information for biodiversity conservation, and can be used to identify action needs. Some of these needs include continued monitoring of species over time by means of informative and accurate methods.

Thailand has used national development plans (i.e., the National Economic and Social Development Plan) as the framework for setting priorities and for resource allocation and conservation since the inception of its first Five-year National Development Plan over 40 years ago [7]. One important goal is to conserve and rehabilitate forest areas for biodiversity conservation, including national parks, wildlife sanctuaries, and watersheds [7]. However, many protected areas in Thailand still require information on the current status of many mammalian populations to move conservation actions forward.

A camera trap survey was conducted in a portion of Thung Yai Naresuan (East) Wildlife Sanctuary, a protected area located on the western national border of Thailand and Myanmar, to estimate the presence and relative abundance of terrestrial mammals. Such surveys are often a cost-effective, less invasive, and less time-consuming method to take inventory of elusive and secretive mammal species, and they have commonly been used to quantify the presence, abundance, and/or density of many terrestrial mammal species, as in [8,9,10,11,12]. Specifically, we used the data from this survey to inventory mammal species composition, to estimate the relative abundance of mammals, and to compare our results with an adjacent sanctuary in order to provide baseline information to formulate conservation and management strategies for endangered species and wildlife habitats in the region. We comment on the promise of this technique for monitoring mammals in the future, and identify ways to augment survey efforts to more effectively conserve species and their habitats.

## 2. Materials and Methods

### 2.1. Study Area

**Figure 1:**
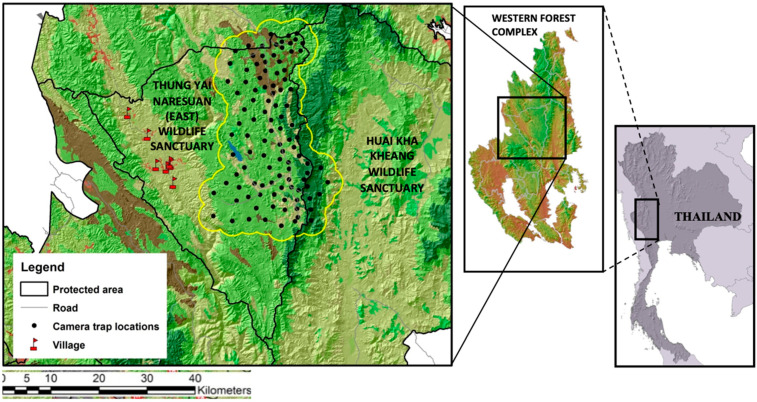
Location of camera trapping array (outlined in yellow) in the Thung Yai Naresuan (East) Wildlife Sanctuary within the Western Forest Complex of Thailand.

The Thung Yai Naresuan Wildlife Sanctuary, established in 1974, is located in the Western Forest Complex (WEFCOM) along the Thailand–Myanmar border (14°55′−15°45′ N, 98°25′−99°05′ E), with an area of 3690 km^2^, is one of Thailand’s largest protected forest areas (Figure 1). Thung Yai Naresuan and the contiguous Huai Kha Khaeng Wildlife Sanctuary (HKK) are located at the crossroads of three plant geographic regions: Indo-Burma, Indo-China, and Indo-Malaya, as well as two zoo-geographic regions: India Indo-China and the Sundaic sub-region [13]. This bio-geographical overlap provides a unique assemblage of Asian species, and it is the key area for terrestrial biodiversity in Thailand [13,14]. Therefore, UNESCO designated these areas, the core of the WEFCOM, as a Natural World Heritage Site in 1991 [13,15,16].

The climate of this region is monsoonal and ranges from tropical to semi-tropical, with a dry season from November to May and a wet season from May to October [17,18]. Annual rainfall in the west is 2000–2400 mm, declining to 1600–2000 mm in the east, with more than 80% of the rain produced by the southwest monsoons [17,18]. Mean minimum and maximum temperatures range from 15 °C to 35 °C during the dry season and 20 °C to 33 °C during the wet season [18,19]. Minimum and maximum night and day temperatures range from 7 °C to 40 °C [19].

Thung Yai Naresuan Wildlife Sanctuary contains a variety of forest types, including mixed deciduous (45%), dry evergreen forest (31%), and hill evergreen forest (15%) [16]. Secondary forests at varying stages of regeneration cover 4% of the sanctuary, and the remaining 5% consists of savanna, deciduous dipterocarp forest, and grassland [16,17,18]. Dry evergreen forests are found at higher altitudes, while mixed deciduous species are found at lower altitudes, where they are usually mixed with bamboo and are located adjacent to streams [16]. The sanctuary is characterized by rugged mountainous terrain, with elevations rising up to 2000 m [16,18]. A small plain zone with grassland and adjacent hills can be found in the northeastern part of the Thung Yai Naresuan East (TYNE) Wildlife Sanctuary, with a moderate altitude zone found in the center of the sanctuary; there is also a mountain range of 1000 m to 2000 m in elevation in the eastern part of the sanctuary [18].

Within the Thung Yai Naresuan Wildlife Sanctuary, this project focused on a 925 km^2^ study area in the north and eastern half of Thung Yai Naresuan East (TYNE; Figure 1). We compared our data with the findings from an adjacent study [20] in the northwest portion of the Huai Kha Khaeng (HKK) Wildlife Sanctuary, just to the east of TYNE. The centers of the two camera arrays were about 35 km apart. Study area elevations in TYNE averaged 967 m (range 664–1335) vs. the average elevations of 200–500 m (range 160–1687 m) in HKK [20], As a consequence of these elevational differences, the TYNE study area likely has a higher proportion of dry evergreen forest vs. more mixed deciduous forest with bamboo in HKK. In addition, there is more protection and less habitat alteration by humans within HKK.

### 2.2. Camera Trapping

We deployed 50 cameras in a grid of 106 trap sites for 15- to 20-day intervals during April 2010–January 2012. Our aim was to photograph tigers (*Panthera tigris*; scientific names of other species are listed in Table 1), their competitors, their prey, and other terrestrial mammals. Two types of digital cameras were used: Stealth Cam I590 with 5.0 megapixels and Bushnell Trophy Cam infrared with 8.0 megapixels. Cameras were set to operate 24 h/day with 3 images per trigger. We used the factory-set 1 min delay for the Stealth Cam cameras and 30 s delay between photo triggers for the Bushnell Trophy Cam, and cameras types were randomly assigned to trap sites. In hindsight, we recognize that camera performance can vary by type, and ideally, we would have used the same camera type and settings at all locations. Each camera was placed on relatively flat terrain 0.4–0.5 m off the ground on a tree, and all vegetation and debris was cleared from the field of view so that both medium-size and large animals had the opportunity to be photographed. In order to obtain adequate numbers of tiger captures and other wildlife species, we placed each camera trap on an animal trail where tiger and leopard signs (scats, scrapes, scent deposits, tracks) or prey signs occurred [8]. Because tigers and leopards were the target species of our efforts, we placed two cameras at each location, opposite each other, to capture simultaneous photos of both sides of the animals. The maximum spacing between trap locations was 3.5 km, and the minimum trap spacing was 2 km. Each trap location was checked on a weekly basis to replace batteries and memory cards, if necessary. The camera trapping efforts in HKK [20] were similar to those in TYNE; two cameras per station were set on trails with animal signs, 30–40 cm off the ground, with 3 photos per trigger. HKK trap stations were set in 1 km^2^ grids (>500 m apart) for 30 days.

We obtained multiple photos of some individuals at the “same” time, either because both cameras at a site worked as intended, or because the cameras were capable of taking multiple photos in a short period of time (<1 min). Thus, to avoid pseudo-replications, we applied the method of Yasuda [21] and considered the first capture of an animal species as an independent record [21,22]. The independence of the detections was defined as (1) consecutive photographs of different individuals of the same species (e.g., individually identifiable species such as tigers and leopards); (2) consecutive photographs of individuals of the same species when separated by more than 30 min; and (3) non-consecutive photos of individuals of the same species, regardless of the time interval [21,23,24]. We defined “photo rate” as the number of independent photographs of each species summed across all camera traps in the study per 100 camera-trap nights [23,25]. The total number of trap-nights was calculated as the sum of all days that individual camera pairs were deployed and at least one of the two cameras was in working order. These data characteristics were similar for the HKK study [20]. Differences in photo rates of species between areas were evaluated with Chi-square tests.

To assess the overall sampling effort, we plotted species accumulation curves for total species and for taxonomic groupings (carnivore, herbivores, and omnivores) against the number of camera trap sample locations and fitted a logarithmic trend line. We then calculated how long it took to record 90% of each taxon we ultimately recorded.

## 3. Results

Overall, 106 camera trap locations in TYNE, sampled for 15–20 days each, resulted in a total of 1817 trap nights. From this effort, 4871 photographs of mammals were obtained, which was reduced to 1821 independent photos based on the 30 min “independence” cut off [26]. Photos were taken of 32 mammal species (Table 1), including 17 IUCN-listed mammal species identified as Endangered or Critically Endangered (*n* = 5), Vulnerable (*n* = 8), and Near Threatened (*n* = 4).

The northern red muntjac (22.5 photos/100 trap-nights (TN)), large Indian civet (19.1), Malayan porcupine (10.6), and sambar deer (10.0) were the most frequently recorded species, representing 62% of all species records (Table 1). The Asiatic jackal, clouded leopard, marbled cat, and Sunda pangolin (listed as Critically Endangered) were the least recorded species (<0.1/100 TN), each of which was photographed on a single occasion. The carnivore community was represented by 6 felids (tiger, leopard, golden cat, clouded leopard, marbled cat, and leopard cat), two canids (dhole and Asiatic jackal), two ursids (Asiatic black bear and Malayan sun bear), and 8 smaller carnivores (Viverridae, Mustelidae, and Herpestidae). Among the other globally endangered species, the Asian elephant was most frequently recorded (4.1/100 TN), followed by Malayan tapirs (1.7), tigers (1.5), and dholes (1.3). Other identified ungulates included the gaur (1.2/100 TN), serow (0.2), lesser mouse deer (0.3), and wild boar (3.5). Only one primate, the stump-tailed macaque, was photographed. Tree shrews and rats were photographed, but their species could not be identified. 

**Figure 2:**
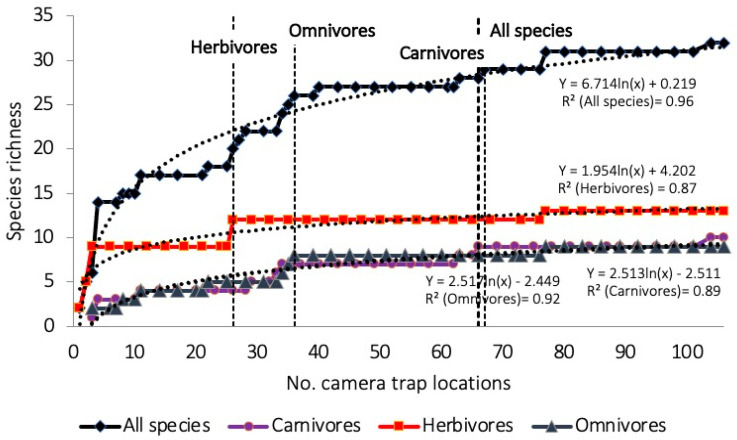
Species accumulation curves for large mammal taxa in Thung Yai Naresuan (East) Wildlife Sanctuary; vertical dash lines demonstrate the number of camera trap locations needed for 90% detection of all large mammal, carnivore, herbivore, and omnivore species.

With our sampling effort of 15–20 days/site, species accumulation curves reached 90% of total species recorded earliest for herbivores (26 sites), slightly later for omnivores (36 sites), and latest for carnivores (66 sites); this threshold was reached at 67 sites for all species in total (Figure 2). We saw no indications that an individual species was found most often in the north or east as a spillover from the neighboring sanctuary.

## 4. Discussion

Based on the extensive list of mammals recorded in TYNE [27], the species we recorded represent only 65% of all mammal species likely to live in this area. This is not surprising, given that our cameras, like in many other such studies focusing on tigers and leopards, were placed near animal trails where signs of large predators and their prey had been observed. Such sites are unlikely to detect the burrowing (e.g., bamboo rat spp.), arboreal (e.g., squirrels, macaques, monkeys, binturong), and aquatic mammals (e.g., otter spp.), much less the volant ones (e.g., bats), that likely occur in our studied area. Our species accumulation curves appeared to level off, indicating the sufficiency of the sampling to detect most species in the area, and this may, indeed, be true given the kind of species which we expected to identify. Still, these limitations of our technique, used by us or others, should continue to be recognized.

In addition, our sampling effort (*n* = 1817 TN, 106 sites, 15–20 days per site) may have been too small to record the presence of very rare species. In comparison with the Huai Kha Khaeng Wildlife Sanctuary (HKK; *n* = 6596 TN, 192 sites, mean = 34 days per site [28]), we recorded 10 fewer species/taxa in TYNE, although we did record three species not recorded in HKK (Table 1). Of these 13 species recorded in only one of the two areas, photo-rates were ≤0.1/100 TN for 7, and ranged from 0.3–0.4/100 TN for 4 others. Thus, these species may have been rare enough such that camera traps were not in place for a long enough period of time to record them (e.g., marbled cat and clouded leopard [28,29,30,31,32]).

No endangered banteng were recorded in TYNE, but had photo-rates of 7.1/100 TN in HKK; we suspect that their disappearance in TYNE can be explained by the high poaching pressure there [33]. In addition, the endangered large spotted civet (0.1/100 TN), and Phaayre’s leaf monkey (<0.1) were recorded in HKK, but not in TYNE. Conversely, no Asiatic elephants were recorded in HKK (vs. 4.1/100 TN in TYNE), though they are known to occur there [34,35]. Other notable disparities between these two adjacent areas were that photo-rates of tigers, leopards, golden jackals, common palm civets, sambar, wild boar, and rat species in HKK were ≥2 times those in TYNE (*X*^2^ = 8.83–367.34, 1 d.f., *p* < 0.003).

More protection and less habitat alteration by humans within HKK, along with the past policy to relocate all hill-tribe villages out of HKK since the 1980s [36,37], have likely resulted in the higher abundance and presence of wildlife there. Additionally, mixed deciduous forest (MDF) is a dominant forest type in the HKK study site and was reported as a major habitat used by the gaur, sambar, barking deer, and wild pig species [38,39]. The intensive use of MDF is probably related to the higher availability of nutritious grass and abundant browse in MDF (more common semi-evergreen forest in the TYNE study area), resulting in high forage quality and quantity and, thus, increased survival of many young herbivores [40]. The result of less human disturbance and good prey abundance in the HKK also supports the abundance of main predators such as the tiger, leopard, jackal, and dhole species, and could be the reason why these carnivores were more abundant there than in TYNE. The previous studies elsewhere also indicated that the areas with high prey abundance and less disturbance are highly suitable for these large predators and have potential for the long-term viability of these large mammals [23,39,41,42,43,44].

Conversely, in TYNE, photo-rates were ≥2 times higher than in HKK for Malayan sun bears, yellow-throated martens, hog badgers, masked palm civets, stump-tailed macaques, Malayan tapirs, and Asiatic brush-tailed porcupines (*X*^2^ = 8.93–237.11, 1 d.f., *p* < 0.0028). Some of these apparent differences may be due to limited sampling, that is, small sample sizes or more limited areas where sampling occurred within parts of each sanctuary, because overall, the areas did not differ much in the proportions of major vegetation types [45,46]. For example, a study of golden jackals (a very widespread species in southern Asia [47]) in a limited portion of HKK indicated that they prefer dry dipterocarp forest compared to other habitat types [46]; thus, their apparent rarity in TYNE (compared to HKK jackal study area) may be due to the relatively limited abundance of dry dipterocarp forest there. For smaller species, the factors affecting their relative abundance were less well known; thus, we were less able identify them using camera-trapping protocols and related analyses.

Finally, we note that within TYNE, our camera grid was limited to the eastern part of the sanctuary, but adjacent to the western part, where local people still practice farming and where other human activities regularly occur. The presence of some larger mammal species, especially, may be more influenced by human proximity and activities in this and other similar areas [2,4,6,12].

Based on our results, the TYNE and the HKK still hold a rich community of mammals compared to the protected areas in other regions in Thailand [14]. This could be the result of being well-protected from human disturbance activities compared to other sites [14]. The exclusion of human activities from protected areas makes these sanctuaries suitable to provide key habitats for maintaining wildlife populations. However, poaching still occurs inside both TYNE and HKK, especially the western TYNE, where local people farm. The demand for land and other resources (wild meat, non-traditional forest products, etc.) could lead to a change the abundance of species in the future [12]. Therefore, landscape scale management based on ecosystem management practices should be taken into account, along with improving protection, strengthening coordination among protected areas, and strengthening conservation awareness in local communities.

## 5. Conclusions

The total number of mammal species documented in this study certainly does not represent the full complement of this area. In part, this was likely because the surveys were somewhat limited in length (but not number of camera sites) and, thus, rarer species were less likely to be identified. Moreover, some species in the area were less likely to be photographed due to behavior, territoriality, size, and specific habitat preferences. Still, the use of camera traps is promising as an index for species abundance because, logically, when density increases, the chances of encounters between individuals and cameras would be expected to increase [22,23,24,48,49,50]. Such surveys are also useful when individual recognition, and, thus, capture–recapture density analysis, is not possible (though using encounter rates from camera trap data as an index of density may be applicable [24,50,51,52,53]). Therefore, although the accuracy of this method should be further evaluated, the index provided by camera trapping is an important survey method for estimating the occurrence and conservation status of many wildlife species, especially for those identified as threatened or endangered. Follow-up studies in this and similar areas can be improved by increasing the duration for which cameras are in the field and adding cameras that target small mammals [54], as well as arboreal [55,56] and semi-aquatic species [57]. If budgetarily prudent, cutting-edge eDNA surveys would also enhance the chances to identify a wider range of species of concern [58]. Still, technological solutions will likely need to be complemented with traditional observer-based methods in many regions [59].

Our surveys indicate that Thung Yai Naresuan (East) Wildlife Sanctuary contains significant populations of mammals that are nationally and globally endangered, such as tigers, dholes, elephants, Malayan tapirs, and Sunda pangolins. The large number of globally threatened mammal species which we recorded confirms that TYNE and adjacent areas provide a center for region-wide conservation and offer long-term prospects for the survival and persistence of an intact large mammal community compared to the other protected areas in other regions of Thailand [14]. The limitation, and even exclusion, of human activities from protected areas makes these sanctuaries more suitable for providing key species-specific habitats for maintaining wildlife populations. However, poaching still occurs in both surveyed areas [14], especially the western TYNE, where local people still farm [25]. Thus, the demand for land and other resources (wild meat, NTFP, etc.) could lead to changes in the abundance of various species in the future. Therefore, landscape-scale management based on ecosystem management practices, including regular monitoring, should be taken into account, along with improving protection, strengthening coordination among protected areas, and strengthening conservation awareness for local communities. With such efforts, the sustainability of this unique tropical ecosystem can be ensured for future generations.

## Figures and Tables

**Table 1:** A comparison of photographic rates (photos/100 trap nights) of mammal species recorded in Thung Yai Naresuan (East) (TYNE) Wildlife Sanctuary in April 2010–March 2012 (*n* = 1817 trap-nights) and in Huai Kha Kheang (HKK) Wildlife Sanctuary in November 2017–March 2020 (*n* = 6596 trap-nights; [21]) in Thailand. IUCN Red Book statuses (CR—Critically Endangered; EN—Endangered; VU—Vulnerable; NT—Near Threatened) are listed in parentheses.

Species	Scientific Name	TYNE	HKK
Tiger (EN)	*Panthera tigris*	1.5	3.7
Leopard (VU)	*Panthera pardus*	2.4	9.7
Golden cat (NT)	*Pardofelis temminckii*	0.5	<0.1
Clouded leopard (VU)	*Neofelis nebulosa*	<0.1	<0.1
Marbled cat (NT)	*Pardofelis marmorata*	<0.1	(np) ^a^
Leopard cat	*Prionailurus bengalensis*	2.2	3.1
Dhole (EN)	*Cuon alpinus*	1.3	1.3
Golden jackal	*Canis aureus*	<0.1	11.9
Asiatic black bear (VU)	*Ursus thibetanus*	0.3	0.5
Malayan sun bear (VU)	*Helarctos malayanus*	0.8	0.3
Crab-eating mongoose	*Herpestes urva*	1.3	1.2
Small Indian mongoose	*Herpestes javanicus*	(np)	<0.1
Yellow-throated marten	*Martes flavigula*	1.1	0.2
Hog badger (VU)	*Arctonyx collaris*	3.7	0.1
Large Indian civet	*Viverra zibetha*	19.1	13.1
Masked palm civet	*Paguma larvata*	3.2	0.4
Banded linsang	*Prionodon linsang*	0.1	<0.1
Common palm civet	*Paradoxurus hermaphroditus*	1.7	8.0
Large-spotted civet (EN)	*Viverra megaspila*	(np)	0.1
Small Indian civet	*Viverricula indica*	0.3	0.4
Binturong	*Arctictis binturong*	(np)	<0.1
Stump-tailed macaque (VU)	*Macaca arctoides*	2.6	0.2
Northern pig-tailed macaque (VU)	*Macaca leonina*	(np)	0.4
Rhesus macaque	*Macaca mulatta*	(np)	0.3
Asiatic elephant (EN)	*Elephas maximus*	4.1	(np)
Gaur (VU)	*Bos gaurus*	1.2	0.7
Banteng (EN)	*Bos javanicus*	(np)	7.1
Sambar (VU)	*Cervus unicolor*	10.0	33.2
Northern red muntjac	*Muntiacus vaginalis*	22.7	13.8
Sumatran serow (VU)	*Capricornis sumatraensis*	0.2	<0.1
Lesser mouse deer	*Tragulus javanicus*	0.3	(np)
Hog deer	*Axis porcinus*	(np)	0.1
Wild boar	*Sus scrofa*	3.5	10.9
Malayan tapir (EN)	*Tapirus indicus*	1.7	0.6
Sunda pangolin (CR)	*Manis javanica*	<0.1	<0.1
Malayan porcupine	*Hystrix brachyura*	10.6	12.2
Asiatic brush-tailed porcupine	*Atherurus macrourus*	3.7	<0.1
Burmese hare	*Lepus peguensis*	(np)	0.3
Tree shrew spp.		<0.1 ^b^	<0.1 ^b^
Rat spp.		0.2 ^b^	1.0 ^b^
Squirrel spp.		(np)	0.1 ^b^
Phayre’s leaf monkey (EN)	*Trachypithecus phayrei*	(np)	<0.1

^a^ No photos obtained. ^b^ Identified and unknown species pooled.

## Data Availability

All relevant, collated data are presented in the paper, and raw data may be available from the senior author, as dictated by government restrictions.
